# No association of breast cancer risk with integrin beta_3_ (*ITGB3*) Leu33Pro genotype

**DOI:** 10.1038/sj.bjc.6602674

**Published:** 2005-06-21

**Authors:** S E Bojesen, A Tybjærg-Hansen, C K Axelsson, B G Nordestgaard

**Affiliations:** 1 Department of Clinical Biochemistry, Herlev University Hospital, Herlev Ringvej 75, Herlev DK-2730, Denmark; 2 The Copenhagen City Heart Study, Bispebjerg University Hospital, Bispebjerg Bakke 23, Copenhagen NV DK-2400, Denmark; 3 Department of Clinical Biochemistry, Rigshospitalet, Copenhagen University Hospital, Blegdamsvej 9, Copenhagen Ø DK-2100, Denmark; 4 Department of Breast Surgery, Herlev University Hospital, Herlev Ringvej 75, Herlev DK-2730, Denmark

**Keywords:** integrins, breast cancer, genetic polymorphism, genetic epidemiology, case–control

## Abstract

To pursue a borderline increased risk of breast cancer for carriers of two integrin beta_3_ (*ITGB3*) 33Pro alleles found in a recent prospective study, we conducted a case–control study of 1088 women with breast cancer and 4815 female controls. Leu33Pro heterozygotes, homozygotes and heterozygotes+homozygotes *vs* noncarriers had odds ratios for breast cancer of 1.0 (95% confidence interval: 0.8–1.1), 0.8 (0.5–1.2) and 1.0 (0.8–1.1), respectively. After stratification for conventional risk factors, odds ratio for breast cancer in heterozygotes, homozygotes and heterozygotes+homozygotes *vs* noncarriers were not increased above 1.0 in any of the 14 strata examined. This was also true after stratification for tumour histological subtype and cancer stage at the time of diagnosis.

In a recent large prospective study of the general population, we demonstrated an increased risk of all cancer among integrin beta_3_ (*ITGB3*) Leu33Pro homozygotes (Bojesen *et al*, 2003). It is unclear as to which cancer subtype(s) account for this; however, subanalyses suggested that this risk increase partly could be caused by an increased risk of invasive breast cancer, ovarian cancer and/or melanoma. Although two later case–control studies did not confirm an increased risk of invasive breast cancer among Leu33Pro homozygotes (Ayala *et al*, 2003; Jin *et al*, 2004), certain considerations nevertheless favour the possibility that the *ITGB3* Leu33Pro polymorphism may influence risk of invasive breast cancer. First, beta_3_ integrins are ectopically expressed on breast carcinoma cells where they enhance invasive and metastasiogenic properties of these cells (Murthy *et al*, 1996; Wewer *et al*, 1997; Chen *et al*, 2001, 2004; Rolli *et al*, 2003). Second, the Pro33 *vs* Leu33 allele enhances integrin-mediated activation of MAPK pathways (Vijayan *et al*, 2003), crucial for the malignant potential of cancer cells (Johnson and Lapadat, 2002). Third, in our previous prospective study there appeared to be a gene–dosage relationship as Leu33Pro heterozygotes and homozygotes *vs* noncarriers had hazard ratios for invasive breast cancer of 1.2 (95% confidence interval: 0.9–1.7) and 1.9 (1.0–3.7).

For this reason, we tested the hypothesis that *ITGB3* Leu33Pro heterozygosity and homozygosity have an increased risk of invasive breast cancer. Given the prospective data, the assumption of a dominant genetic effect seems reasonable, so we also combined the heterozygous and the homozygous participants in one group *vs* noncarrier participants. We recruited 1088 consecutive female patients with invasive breast cancer and compared these with 4815 women from the general population without breast cancer. This large case–control study also allowed us to search for genotype-associated increased risk of breast cancer in women stratified for conventional risk factors, and to determine whether genotype-associated breast cancer risk is restricted to certain histological subtypes or cancer stages.

## MATERIALS AND METHODS

### Study populations

Cases of invasive breast cancer in women (*n*=1088) were consecutively collected from the Department of Breast Surgery, Herlev University Hospital from 2001 to 2004. Owing to centralised breast cancer care, this department covers all cases of invasive breast cancer in roughly half of the Greater Copenhagen area, a population amounting to 320 000 women the 1st of January 2004. All patients filled in a questionnaire on lifestyle and health, including reproductive, menstrual and hormonal history, alcohol consumption, height and weight. Premenopausal women were defined as women who had a menstrual period within the previous 2 months. Postmenopausal women had a period more than 12 months ago. If the menstrual period was 2–12 months, the level of follicular-stimulating hormone decided whether the patient was pre- or postmenopausal. Furthermore, women >55 years of age after hysterectomy or with menstruation on hormonal substitution were considered postmenopausal. One or more questionnaire informations were missing for 546 women. However, for test of the main hypothesis (genotype association with risk of breast cancer adjusted for age), full information was available on all 1088 breast cancer cases.

Histological classification of tumours according to the WHO classification (Scarff and Torloni, 1968) and histological grading according to Bloom and Richardson (1957) was performed by pathologists blinded to patient genotype. Grade of malignancy of tumours with ductal histology was scored according to Scarff, Bloom and Richardson (Bloom and Richardson, 1957; Scarff and Torloni, 1968). Oestrogen and progesterone receptor status were determined with immunohistochemistry. An examination was positive if at least 10% of the nuclei in malignant tissue presented with typical staining for oestrogen and/or progesterone receptors. Owing to delay in registration, we did not have information on all these characteristics for 278 women.

The controls were women recruited from the other half of the Greater Copenhagen area from the 3rd examination (1991–94) of the Copenhagen City Heart Study (Appleyard *et al*, 1989; Schnohr *et al*, 2001). Age and other covariates used in the statistical models were recorded at this examination. Cases of breast cancer registered until the end of 2002 in the Danish Cancer Registry were excluded from the controls. In both cases and controls, >97% were whites of Danish descent.

### Ethical approvals

All participants gave written informed consent. The scientific ethical committee of Copenhagen and Frederiksberg approved the collection of controls (No. 100.2039/91), and the scientific ethical committee of the County of Copenhagen approved the collection of cases (KA 02152). The studies were also approved by Herlev University Hospital.

### Genotyping

Participants were genotyped as described earlier (Zimrin *et al*, 1990). In short, the Leu33Pro polymorphism is a T → C substitution in exon 3 at position 176 in the beta_3_ integrin gene (GenBank Accession no. NM000212.1), which introduces an *Msp*I recognition site. The assay also included a second *Msp*I recognition site always cleaved, which served as a control site for the digestion reaction. A 268 bp fragment of exon 3 was amplified from genomic DNA with flanking intronic primers, cleaved with *Msp*I, run on a 3% agarose gel and visualised by staining with ethidium bromide. Genotypes were determined independently by an experienced lab technician and an author (SEB).

### Statistical analysis

The statistical software package STATA (2004) was used. All statistical tests were two-sided. *P*<0.05 was considered statistically significant. We used Mann–Whitney *U*-test and Pearson's *χ*^2^ test. Correction for multiple comparisons was by the Bonferoni method. We used NCSS 2001 and PASS 2000 power calculation software (Hintze, 2001) and a logistic regression power analysis to calculate the statistical power of this study.

We performed a matched case–control study with strata of 1, 3 and 5 years of age. This resulted in 63 one-year strata with a mean of 4.5 controls per case, 22 three-year strata and 4.5 controls per case and 14 five-year strata and 4.6 controls per case, respectively. Using 1-year strata, two cases both aged 93 were unmatched with controls and 168 controls aged less than 28 were unmatched with cases. Conditional logistic regression was used to calculate odds ratio for invasive breast cancer according to genotype. Two models were applied: an age-matched model and an age-matched model also adjusted multifactorially for body mass index (continuous), alcohol consumption (continuous), parity (0 *vs* more childbirths), use of oral contraceptive pill (yes/no), menopausal status (pre- *vs* postmenopausal) and use of hormonal replacement therapy (yes/no). In the analysis of histological subtype and invasive breast cancer stage, each subtype/stage was compared to the controls with the age-matched model of 1-year age strata while excluding cases without the analysed subtype/stage.

## RESULTS

Characteristics of participants are shown in Table 1. In cases and controls, 768 (71%) and 3385 (70%) were noncarriers, 296 (27%) and 1300 (27%) were heterozygotes and 24 (2%) and 130 (3%) were homozygotes, respectively. These genotype distributions were both in Hardy–Weinberg equilibrium (*P*=0.67 and 0.70).

Age-matched odds ratios for breast cancer in heterozygotes, homozygotes and heterozygotes+homozygotes *vs* noncarriers were 1.0 (95% confident interval: 0.8–1.1), 0.8 (0.5–1.2) and 1.0 (0.8–1.1), respectively (Table 2). Identical analyses with age strata of 3 and 5 years for matching of cases and controls gave similar results. Multifactorial adjustment did not substantially change these odds ratios. We had 90% statistical power to detect odds ratios equal to or larger than 1.3, 1.8 and 1.3 in heterozygous, homozygous and heterozygous+homozygous *vs* noncarriers, respectively.

**Figure 1:**
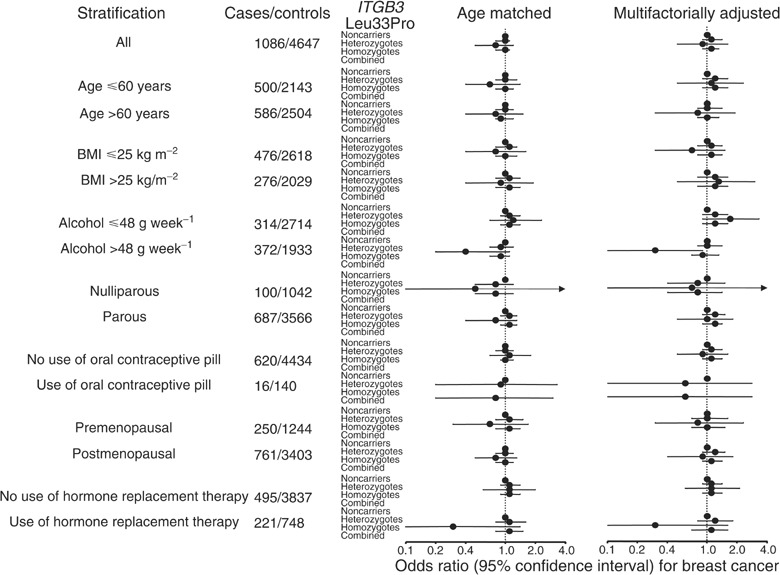
Risk of invasive breast cancer by *ITGB3* Leu33Pro genotype. Number of cases/controls refer to the 1-year age strata calculations and vary through stratifications due to incomplete information on the stratification in question. Multifactorial adjustment was for body mass index (continuous), alcohol consumption (continuous), parity (0 *vs* more childbirths), use of oral contraceptive pill (yes/no), menopausal status (pre- *vs* postmenopausal) and use of hormone replacement therapy (yes/no).

To explore whether genotype interacts with conventional risk factors for breast cancer, we stratified the age-matched and multifactorially adjusted analyses by age, body mass index, alcohol consumption, parity, use of oral contraceptive pills, menopausal status and use of hormonal replacement therapy. Risk of breast cancer in homozygotes, heterozygotes and heterozygous+homozygous *vs* noncarriers was not increased above 1.0 in any of the 14 subgroups of women examined (Figure 1). Although the adjusted odds ratio in homozygotes *vs* noncarriers among women with alcohol consumption >48 g week^−1^ was 0.3 (0.1–0.9), this association became nonsignificant after correcting for multiple comparisons. Furthermore, this association was statistically nonsignificant in the age-matched study with more statistical power.

Risk of breast cancer in homozygotes, heterozygotes and heterozygous+homozygous *vs* noncarriers did not differ from 1.0 in any histological subgroup of invasive breast cancer Table 3.

Risk of invasive breast cancer in homozygotes, heterozygotes and heterozygous+homozygous *vs* non-carriers did not differ from 1.0 in any cancer stage subgroup (Table 4).

## DISCUSSION

So far, four papers have been published on this polymorphism and breast cancer risk (Ayala *et al*, 2003; Bojesen *et al*, 2003; Jin *et al*, 2004; Wang-Gohrke and Chang-Claude, 2004). Our own first study was explorative in nature and found a hazard ratio in heterozygotes and homozygotes *vs* noncarriers of 1.2 (0.9–1.6) and 1.9 (1.0–3.7), *P*-value 0.06, so this could represent a chance finding. In contrast to another study, Ayala *et al* (2003) reduced risk of breast cancer in heterozygotes *vs* noncarriers in a case–control study of 100 cases and 100 controls. However, genotype distribution among controls was not in Hardy–Weinberg equilibrium (*P*=0.048) and the finding was only significant at *P*=0.04, so this also could represent a chance finding. In a third study (Jin *et al*, 2004), no increased (or decreased) risk was found in heterozygotes or homozygotes. In the fourth study (Wang-Gohrke and Chang-Claude, 2004) of 602 cases and 1054 controls, the odds ratios for heterozygotes, homozygotes and heterozygotes+homozygotes *vs* noncarriers were 1.1 (0.9–1.4), 1.3 (0.7–2.4) and 1.1 (0.9–1.4), respectively. A borderline (*P*=0.055) interaction between age (age ⩽45 *vs* >45 years) and genotype (noncarriers *vs* heterozygotes+homozygotes) with an odds ratio of heterozygotes+homozygotes *vs* noncarriers of 1.3 (1.0–1.8) in women aged over 44 years; in our study, no such interaction could be detected (*P*=0.64). They also detected no association between tumour stage or grade and genotype (noncarrier *vs* heterozygous+homozygous state) after correcting for multiple comparisons.

Like the previous studies mentioned, the present study has certain limitations. Covariates for controls were recorded at the 1991–1994 examination, while those for the cases were recorded at the time of diagnosis in 2001–2004, approximately 10 years apart. For conventional risk factors such as the use of hormonal replacement therapy, this could distort risk estimations, but will not invalidate analyses of genetic risk factors, as genotypes are permanent. Although cases and controls were recruited from slightly different geographical locations of the greater Copenhagen area, the background population is the same, more than 97% being white women of Danish descent in both cases and controls.

Overall, the combined evidence from this and previous reports indicates that *ITGB3* Leu33Pro heterozygosity and homozygosity do not increase the risk of breast cancer.

Future research should no longer focus on risk of breast cancer in *ITGB3* Leu33Pro homozygotes, but should rather try to examine other cancer subgroups like ovarian cancer, melanoma or yet other cancer subgroups (Bojesen *et al*, 2003), as explanations for the increased overall cancer risk in Leu33Pro homozygotes.

## Figures and Tables

**Table 1:** Characteristics of participants

	**Invasive breast cancer**	**Controls**	***P*-value**
Number	1088	4815	—
Age (years)	62 (29–93)	61 (22–90)	<0.0001
Body mass index (kg m^−2^)	24 (14–51)	24 (13–50)	0.0001
Alcohol consumption (g week^−1^)	48 (0–720)	36 (0–960)	0.0002
Nulliparous (%)	13 (11–16)	25 (24–26)	<0.0001
Use of oral contraceptive pill (%)	3 (1–4)	4 (4–5)	0.02
Postmenopausal (%)	76 (73–79)	71 (69–72)	0.0009
Use of hormonal replacement therapy (%)	31 (27–34)	16 (15–17)	<0.0001

Values represent median (range) or frequencies (95% confidence interval).

*P*-values are for invasive breast cancer *vs* controls on Mann–Whitney *U*-test or Pearson's *χ*^2^ test.

**Table 2:** Risk of invasive breast cancer by *ITGB3* Leu33Pro genotype

			**Odds ratio (95% CI)**			
	**Strata**	**Cases/controls**	**Noncarriers 33Leu/Leu**	**Heterozygotes 33Leu/Pro**	**Homozygotes 33Pro/Pro**	**Heterozygotes and homozygotes combined**
Age matched	1 year	1086/4647	1.0	1.0 (0.8–1.1)	0.8 (0.5–1.2)	1.0 (0.8–1.1)
	3 years	1086/4715	1.0	1.0 (0.8–1.1)	0.8 (0.5–1.3)	1.0 (0.8–1.1)
	5 years	1088/4755	1.0	1.0 (0.8–1.1)	0.8 (0.5–1.2)	1.0 (0.8–1.1)
90% power to detect^a^			—	1.3	1.8	1.3
Multifactorially adjusted^b^	1 year	540/4397	1.0	1.1 (0.9–1.4)	0.9 (0.5–1.6)	1.1 (0.9–1.3)
	3 years	540/4397	1.0	1.1 (0.9–1.4)	1.0 (0.5–1.7)	1.1 (0.9–1.3)
	5 years	540/4455	1.0	1.1 (0.9–1.4)	0.9 (0.5–1.6)	1.1 (0.9–1.3)
90% power to detect^a^			—	1.4	2.1	1.4

*ITGB3*=integrin beta_3_; CI=confidence interval.

a The smallest odds ratios our study (with 1-year age strata) could detect with 90% statistical power and *P*<0.05 on two-sided tests.

b Including body mass index (continuous), alcohol consumption (continuous), parity (0 *vs* more childbirths), use of oral contraceptive pill (yes/no), menopausal status (pre- *vs* postmenopausal), use of hormone replacement therapy (yes/no).

**Table 3:** Risk of invasive breast cancer by *ITGB3* Leu33Pro genotype stratified for histological subtype

	**Noncarriers 33Leu/Leu**		**Heterozygotes 33Leu/Pro**		**Homozygotes 33Pro/Pro**		**Heterozygotes and homozygotes combined**	
**Histological subtype**	***n* (%)**	**Odds ratio**	***N* (%)**	**Odds ratio (95%CI)**	***N* (%)**	**Odds ratio (95%CI)**	***N* (%)**	**Odds ratio (95%CI)**
Ductal	457 (70)	1.0	180 (28)	1.0 (0.8–1.2)	14 (2)	0.7 (0.4–1.3)	194 (30)	1.0 (0.8–1.2)
Lobular	72 (68)	1.0	32 (30)	1.2 (0.8–1.8)	2 (2)	0.7 (0.2–2.9)	34 (32)	1.1 (0.7–1.7)
Others	37 (76)	1.0	12 (24)	0.8 (0.4–1.6)	0 (0)	—	12 (24)	0.8 (0.4–1.5)
Unknown	202 (72)	1.0	70 (25)	0.9 (0.7–1.2)	8 (3)	0.9 (0.4–2.0)	78 (28)	0.9 (0.7–1.2)
All	768 (71)	1.0	294 (27)	1.0 (0.8–1.1)	24 (2)	0.8 (0.5–1.2)	318 (29)	1.0 (0.8–1.1)

*ITGB3*=integrin beta_3_; CI=confidence interval.

Odds ratios were *vs* 4647 controls and matched for age in one-year strata.

**Table 4:** Risk of invasive breast cancer by *ITGB3* Leu33Pro genotype stratified for characteristics of tumour or dissemination at diagnosis

	**Noncarriers 33Leu/Leu**		**Heterozygotes 33Leu/Pro**		**Homozygotes 33Pro/Pro**		**Heterozygotes and homozygotes combined**	
	***n* (%)**	**Odds ratio**	***n* (%)**	**Odds ratio (95%CI)**	***n* (%)**	**Odds ratio (95%CI)**	***n* (%)**	**Odds ratio (95%CI)**
*Tumour size (mm)*								
⩽10	99 (70)	1.0	43 (29)	1.1 (0.8–1.6)	1 (1)	0.2 (0.0–1.7)	44 (31)	1.0 (0.7–1.4)
11–20	217 (68)	1.0	95 (30)	1.1 (0.9–1.4)	5 (2)	0.6 (0.2–1.4)	100 (32)	1.1 (0.8–1.4)
21–30	163 (73)	1.0	53 (24)	0.8 (0.6–1.2)	8 (4)	1.2 (0.6–2.5)	61 (27)	0.9 (0.6–1.2)
31–50	62 (68)	1.0	27 (30)	1.2 (0.7–1.9)	2 (2)	0.9 (0.2–3.6)	29 (32)	1.2 (0.7–1.8)
>50	26 (76)	1.0	8 (24)	0.8 (0.4–1.9)	0 (0)	—	8 (24)	0.8 (0.3–1.7)
Unknown	201 (73)	1.0	68 (25)	0.9 (0.7–1.2)	8 (3)	0.9 (0.4–2.0)	76 (27)	0.9 (0.7–1.2)

*Grade of malignancy (only ductal histology)*								
Grade I	109 (66)	1.0	52 (31)	1.2 (0.8–1.7)	5 (3)	1.1 (0.4–2.7)	57 (34)	1.2 (0.8–1.6)
Grade II	226 (72)	1.0	83 (26)	1.0 (0.7–1.2)	6 (2)	0.7 (0.3–1.5)	89 (28)	0.9 (0.7–1.2)
Grade III	110 (72)	1.0	40 (26)	0.9 (0.7–1.4)	3 (2)	0.6 (0.2–2.0)	37 (27)	0.9 (0.6–1.3)
Unknown	12 (71)	1.0	5 (29)	1.1 (0.4–3.1)	0 (0)	—	5 (29)	0.9 (0.3–2.7)

*Hormone receptor status*								
Negative	95 (71)	1.0	36 (27)	1.0 (0.7–1.5)	2 (2)	0.4 (0.1–1.7)	38 (29)	0.9 (0.6–1.4)
Positive	449 (69)	1.0	186 (29)	1.1 (0.9–1.3)	13 (2)	0.7 (0.4–1.3)	199 (31)	1.0 (0.9–1.2)
Unknown	224 (73)	1.0	72 (24)	0.8 (0.6–1.1)	9 (3)	1.0 (0.5–2.0)	81 (27)	0.8 (0.6–1.1)

*Lymph node involvement*								
No tumour positive nodes	278 (71)	1.0	104 (26)	1.0 (0.8–1.2)	11 (3)	1.0 (0.5–1.8)	115 (29)	1.0 (0.8–1.2)
Tumour-positive nodes, without breakthrough of capsule	154 (73)	1.0	56 (26)	0.9 (0.7–1.3)	2 (1)	0.3 (0.1–1.2)	58 (27)	0.9 (0.6–1.2)
Tumour-positive nodes, with breakthrough of capsule	128 (67)	1.0	61 (32)	1.2 (0.9–1.7)	3 (2)	0.6 (0.2–1.9)	64 (33)	1.2 (0.9–1.6)
Unknown	208 (72)	1.0	73 (25)	0.9 (0.7–1.2)	8 (3)	0.9 (0.4–1.9)	81 (28)	0.9 (0.7–1.2)

*Distant metastases*								
Absent	535 (70)	1.0	212 (28)	1.0 (0.9–1.2)	16 (2)	0.7 (0.4–1.2)	228 (30)	1.0 (0.8–1.2)
Present	4 (67)	1.0	2 (33)	1.3 (0.2–7.3)	0 (0)	—	2 (33)	1.2 (0.2–6.7)
Unknown	229 (72)	1.0	80 (25)	0.9 (0.7–1.2)	8 (3)	0.8 (0.4–1.7)	88 (28)	0.9 (0.7–1.1)

*ITGB3*=integrin beta_3_; CI=confidence interval.

Odds ratios were *vs* 4647 controls and matched for age in one-year strata.
